# Supplementary material for: Burden of tuberculosis in underserved populations in South Africa: A systematic review and meta-analysis
Source: PLOS Glob Public Health. 2024 Oct 3;4(10):e0003753. doi: 10.1371/journal.pgph.0003753 (PMC11449336; doi:10.1371/journal.pgph.0003753)
Supplement: S1 Table — (DOCX) [file pgph.0003753.s002.docx]

## **S1 Table**. Search Term

| **Table A in S1 Table. Lens.org**  Searched on 06.26.2023  *We hereby want to acknowledge the use of The Lens:* [*https://www.lens.org/*](https://www.lens.org/)*.*  *Restrictions: Years 2010-Jul 26 2023* | | | |
| --- | --- | --- | --- |
| **Search component** | **Search terms** | **Rationale** | **Results** |
| **#1 Tuberculosis** | (mesh_term.mesh_heading: ("Tuberculosis" OR "Latent Tuberculosis" OR  "Tuberculosis, Extrapulmonary" OR "Tuberculosis, Miliary" OR  "Tuberculosis, Multidrug-Resistant" OR "Extensively Drug-Resistant Tuberculosis"  OR "Tuberculosis, Pulmonary" OR "Silicotuberculosis") OR  title: ("tuberculosis" OR "TB") OR  abstract: ("tuberculosis" OR "TB") OR  keyword: ("tuberculosis" OR "TB")) | Search for TB/Tuberculosis in…   - MeSH terms (no explosion possible) - Titles - Abstracts - Keywords |  |
| **#2 Epidemiology** | (mesh_term.mesh_heading: ("Epidemiology" OR "Molecular Epidemiology" OR  "Morbidity" OR "Basic Reproductive Number" OR "Incidence" OR "Prevalence") OR  title: ("prevalence" OR "incidence" OR "epidemiolog*" OR "surveillance") OR  abstract: ("prevalence" OR "incidence" OR "epidemiolog*" OR "surveillance") OR  keyword: ("prevalence" OR "incidence" OR "epidemiolog*" OR "surveillance")) | Search for epidemiology in…   - MeSH terms (no explosion possible) - Titles - Abstracts - Keywords |  |
| **#3 South Africa** | (mesh_term.mesh_heading: ("South Africa") OR  title: ("south africa" OR "south african") OR  abstract: ("south africa" OR "south african") OR  keyword: ("south africa" OR "south african") OR  source.title: ("south africa*") OR  fulltext: ("Cape Town" OR "Soweto" OR "Kathlehong" OR "Umlazi" OR "Soshanguve" OR "Khayelitsha" OR "Mamelodi" OR "Mitchells Plain" OR "Ibhayi" OR "Sebokeng" OR "Mangaung" OR "Philippi" OR "Ivory Park" OR "Botshabelo" OR "Alexandra" OR "Phoenix" OR "KwaMashu" OR "Vosloorus" OR "Mdantsane" OR "Delft" OR "Etwatwa" OR "Motherwell" OR "Tsakane" OR "Thabong" OR "Evaton" OR "Daveyton" OR "Ntuzuma" OR "Madadeni" OR "Embalenhle" OR "Kagiso" OR "Mabopane" OR "Galeshewe" OR "KwaNobuhle" OR "Saulsville" OR "Jouberton" OR "Thokoza" OR "KwaThema" OR "Guguletu" OR "Diepsloot" OR "Ga-Rankuwa" OR "Seshego" OR "Edentale" OR "Osizweni" OR "Orange Farm" OR "Hlubi" OR "Duduza" OR "Mfuleni" OR "Mpumalanga" OR "Matsulu" OR "Thembalethu" OR "Mahwelereng" OR "Sharpeville") OR  fulltext: ("Eastern Cape" OR "Free State" OR "Gauteng" OR "KwaZulu-Natal" OR "Limpopo" OR "Mpumalanga" OR "Northern Cape" OR "North West" OR "Western Cape")) | Search for South Africa in…   - MeSH terms - Titles - Abstracts - Keywords - Journal names - Fulltexts (provinces and townships) |  |
| **#4 Underserved populations** | (mesh_term.mesh_heading: ("Socioeconomic Factors" OR "Economic Factors"  OR "Economic Stability" OR "Economic Instability" OR "Economic Status" OR  "Poverty" OR "Poverty Areas" OR "Social Class" OR "Low Socioeconomic Status"  OR "Social Mobility" OR "Social Factors" OR "Resource-Limited Settings") OR  title: ("informal settlement*" OR "settlement*" OR "informal dwelling*" OR "resource-limited" OR "impoverished" OR "township*" OR "peri-urban") OR  abstract: ("informal settlement*" OR "settlement*" OR "informal dwelling*" OR "resource-limited" OR "impoverished" OR "township*" OR "peri-urban") OR  keyword: ("informal settlement*" OR "settlement*" OR "informal dwelling*" OR "resource-limited" OR "impoverished" OR "township*" OR "peri-urban")) | Search for informal settlement in…   - MeSH terms (no explosion possible) - Titles - Abstracts - Keywords |  |
| **#5 Final search** | **#1 AND #2 AND #3 AND #4** |  | **174** |
| **Table B in S1 Table . EMBASE Ovid (access via Yale University)**  Searched on 06.26.2023  *We hereby want to acknowledge the use of EMBASE Ovid:* [*https://library.medicine.yale.edu/find/title/embase-ovid*](https://library.medicine.yale.edu/find/title/embase-ovid)*.*  *Restrictions: Years 2010-Jul 26 2023* | | | |
| **Search component** | **Search terms** | **Rationale** | **Results** |
| **#1 Tuberculosis** | ((exp tuberculosis/ OR tuberculosis.mp. OR TB.mp.) | Search for TB/Tuberculosis in…   - Emtree terms (EMBASE subject headings) - Keywords |  |
| **#2 Epidemiology** | (exp epidemiological monitoring/ OR exp epidemiological surveillance/ OR  exp epidemiology/ OR epidemiolog*.mp. OR prevalence.mp. OR  incidence.mp. OR surveillance.mp.) | Search for epidemiology in…   - Emtree terms (EMBASE subject headings) - Keywords |  |
| **#3 South Africa** | (exp South Africa/ OR south africa*.mp.) | Search for South Africa in…   - Emtree terms (EMBASE subject headings) - Keywords |  |
| **#4 Underserved populations** | (exp socioeconomics/ OR settlement*.mp. OR informal dwelling*.mp. OR resource-limited.mp. OR impoverished.mp. OR township*.mp. OR peri-urban.mp.)) | Search for informal settlements in…   - Emtree terms (EMBASE subject headings) - Keywords |  |
| **#5 Final search** | **#1 AND #2 AND #3 AND #4** |  | **597** |
| **Table C in S1 Table African Index Medicus**  Searched on 06.26.2023  *We hereby want to acknowledge the use of African Index Medicus:* [*https://www.globalindexmedicus.net/biblioteca/aim/*](https://www.globalindexmedicus.net/biblioteca/aim/)*.*  *Restrictions: None (limited search functionality of database)* | | | |
| **Search component** | **Search terms** | **Rationale** | **Results** |
| **#1 Tuberculosis** | (tw:(tuberculosis OR TB )) | Search for TB/Tuberculosis in…   - Keywords |  |
| **#2 Epidemiology** | (tw:(epidemiolog* OR prevalence OR incidence OR surveillance)) | Would have narrowed down results to only 9. |  |
| **#3 South Africa** | Not searched | Search for South Africa in…   - Keywords |  |
| **#4 Underserved populations** | Not searched | Would have narrowed down results to only 3. |  |
| **#5 Final search** | **#1 AND #3** |  | **33** |
| **Table D in S1 Table . Incidence & Prevalence Database**  Searched on 06.26.2023  *We hereby want to acknowledge the use of the Incidence & Prevalence Database by Clarivate:* [*https://www.tdrdata.com/ipd/ipd_init*](https://www.tdrdata.com/ipd/ipd_init)*.*  *Restrictions: Years 2010-Jul 26 2023* | | | |
| **Search component** | **Search terms** | **Rationale** | **Results** |
| **#1 Tuberculosis** | (tw:(tuberculosis OR TB)) | Search for TB/Tuberculosis in…   - Keywords - Textwords |  |
| **#2 Epidemiology** | Not searched | Limited search function |  |
| **#3 South Africa** | South Africa | Search for South Africa in…   - Country/geographic region |  |
| **#4 Underserved populations** | Not searched | Limited search function |  |
| **#5 Final search** | **#1 AND #3** |  | **0*** |

## ***Legend****: *The search returned several results, but none of them were sufficiently applicable to the research question to add them to the screening process.*
