# Supplementary material for: Burden of tuberculosis in underserved populations in South Africa: A systematic review and meta-analysis
Source: PLOS Glob Public Health. 2024 Oct 3;4(10):e0003753. doi: 10.1371/journal.pgph.0003753 (PMC11449336; doi:10.1371/journal.pgph.0003753)
Supplement: S2 Table — (DOCX) [file pgph.0003753.s003.docx]

## **S2 Table**. Data Extraction Form

| **Section # Item #** | | | **Question type** | **Answer options** |
| --- | --- | --- | --- | --- |
| **General study details** | #1 Study ID  #2 DOI  #3 Title  #4 First author  #5 Publication year  #6 Language of publication  #7 Publication status  #8 Journal | | #1 Short answer  #2 Short answer  #3 Short answer  #4 Short answer  #5 Short answer  #6 Drop-down  #7 Drop-down  #8 Short answer | #1 NA  #2 NA  #3 NA  #4 NA  #5 NA  #6 English, French, Other  #7 Peer-reviewed, Pre-print  #8 NA |
|  |  |  |  |  |
| **Study methods** | #1 Study design  #2 Study setting/site  #3 Individual/household level data  #4 Follow-up period  #5 Study period | | #1 Drop-down  #2 Checkboxes  #3 Multiple choice  #4 Short answer  #5 Short answer | #1 Prospective cohort, retrospective cohort, case-control, cross-sectional, non-randomized, RCT, other  #2 Hospital/inpatient, outpatient, home, other  #3 Individual data, household data  #4 NA  #5 NA |
|  |  |  |  |  |
| **Primary outcome** | #1 Primary outcome  #2 TB case definition | | #1 Multiple choice  #2 Short answer | #1 TB prevalence, TB incidence, LTBI prevalence, LTBI incidence  #2 NA |
|  |  |  |  |  |
| **Primary outcome ascertainment**  **(TB)** | #1 Diagnostic tool  #2 Diagnostic tool characteristics  #3 Prevalence (by HIV status)  #4 Incidence (by HIV status)  #5 Sample size (by HIV status)  #6 TB cases (by HIV status)  #7 Comments | | #1 Checkboxes  #2 Short answer  #3 Short answer  #4 Short answer  #5 Short answer  #6 Short answer  #7 Short answer | #1 Automated NAAT, TB-LAMP, LF-LAM, sputum smear microscopy, bacterial culture, self-report, other  #2 NA  #3 NA  #4 NA  #5 NA  #6 NA  #7 NA |
|  |  |  |  |  |
| **Primary outcome ascertainment**  **(LTBI)** | #1 Diagnostic tool  #2 Diagnostic tool characteristics  #3 Prevalence (by HIV status)  #4 Incidence (by HIV status)  #5 Sample size (by HIV status)  #6 TB cases (by HIV status)  #7 Comments | | #1 Checkboxes  #2 Short answer  #3 Short answer  #4 Short answer  #5 Short answer  #6 Short answer  #7 Short answer | #1 TST, IGRA, other  #2 NA  #3 NA  #4 NA  #5 NA  #6 NA  #7 NA |
|  |  |  |  |  |
| **Study population** | #1 Exposure definition  #2 Exposure definition  #3 Age group  #4 Population age  #5 Population sex/gender  #6 Population race/ethnicity  #7 Comorbidities: PWH  #8 Comorbidities: Diabetes  #9 Comorbidities: Other immunocompromised  #10 Comorbidities: PWID | | #1 Multiple choice  #2 Short answer  #3 Multiple choice  #4 Short answer  #5 Short answer  #6 Short answer  #7 Short answer  #8 Short answer  #9 Short answer  #10 Short answer | #1 Neonates, infants, children, adolescents, adults, other age range  #2 NA  #3 NA  #4 NA  #5 NA  #6 NA  #7 NA  #8 NA  #9 NA  #10 NA |

### ***Abbreviations****: NA = Not applicable; NAAT = Nucleic Acid Amplification Test; LAMP = Loop-mediated Isothermal Amplification; LAM = Lipoarabinomannan; TST = Tuberculin Skin Test; IGRA = Interferon-Gamma-Release Assay; PLWH = People Living With HIV; PWID = People Who Inject Drugs.*
