# Supplementary material for: Burden of tuberculosis in underserved populations in South Africa: A systematic review and meta-analysis
Source: PLOS Glob Public Health. 2024 Oct 3;4(10):e0003753. doi: 10.1371/journal.pgph.0003753 (PMC11449336; doi:10.1371/journal.pgph.0003753)
Supplement: S3 Table — (DOCX) [file pgph.0003753.s004.docx]

## **S3 Table**. Risk-of-Bias Assessment Form[2]

| **Item # Question #** | | | **Answer options** | **Key considerations** |
| --- | --- | --- | --- | --- |
| **Sample frame** | #1 Was the sample frame appropriate to address the target population? | | Yes  No  Unclear  NA | - Are important population characteristics of the sample (e.g., age range, gender, morbidities, other potentially influential factors) reflected in the target population? |
|  |  |  |  |  |
| **Study participants** | #2 Were study participants sampled in an appropriate way? | | Yes  No  Unclear  NA | - Was random sampling (census, cluster sampling) or convenience sampling employed? |
|  |  |  |  |  |
| **Sample size** | #3 Was the sample size adequate? | | Yes  No  Unclear  NA | - Was a sample size calculation conducted? - Is the calculated sample size sufficient to answer the research question? |
|  |  |  |  |  |
| **Study subjects and setting** | #4 Were the study subjects and setting described in detail? | | Yes  No  Unclear  NA | - Are characteristics of the study sample (e.g., sociodemographic variables) described in sufficient detail? |
|  |  |  |  |  |
| **Data analysis** | #5 Was the data analysis conducted with sufficient coverage of the identified sample? | | Yes  No  Unclear  NA | - Is there evidence of coverage bias? |
|  |  |  |  |  |
| **Identification of the condition** | #6 Were valid methods used for the identification of the condition? | | Yes  No  Unclear  NA | - Is there evidence of measurement or classification bias? - Is there evidence of over- or under-reporting? |
|  |  |  |  |  |
| **Measurement of the condition** | #7 Was the condition measured in a standard, reliable way for all participants? | | Yes  No  Unclear  NA | - Validity of the measurement instrument and the performance of the measurements (e.g, was staff trained? Was the measurement standardized?) - Were results obtained by different observers compared? |
|  |  |  |  |  |
| **Statistical analysis** | #8 Was there appropriate statistical analysis? | | Yes  No  Unclear  NA | - Were numerators/denominators, percentages and confidence intervals clearly reported? - Were all measured variables defined? - Was the statistical strategy appropriate? |
|  |  |  |  |  |
| **Response rate** | #9 Was the response rate adequate, and if not, was the low response rate managed appropriately? | | Yes  No  Unclear  NA | - Was the response rate adequate? - Were low response rates and their potential implications discussed? |

### ***Abbreviations****: NA = Not applicable.*

[2] JBI. (2017). *Checklist for Prevalence Studies*. <https://jbi.global/sites/default/files/2019-05/JBI_Critical_Appraisal-Checklist_for_Prevalence_Studies2017_0.pdf>
