# Supplementary material for: Burden of tuberculosis in underserved populations in South Africa: A systematic review and meta-analysis
Source: PLOS Glob Public Health. 2024 Oct 3;4(10):e0003753. doi: 10.1371/journal.pgph.0003753 (PMC11449336; doi:10.1371/journal.pgph.0003753)
Supplement: S4 Table — (DOCX) [file pgph.0003753.s005.docx]

## **Table A in S4 Table**. Risk-of-Bias Assessment of Included Studies

| **Study design** | **Study**  **ID** | **Author**  **[Year]** | **Sample frame** | **Sampling** | **Sample size** | **Study subjects** | **Data analysis** | **Identification of condition** | **Measurement of condition** | **Statistical analysis** | **Response rate** | **Final appraisal*** |
| --- | --- | --- | --- | --- | --- | --- | --- | --- | --- | --- | --- | --- |
| **TB PREVALENCE** | | | | | | | | | | | | |
| Cross-sectional study | 80 | Cox  [2010] | **No** | **No** | Unclear | Yes | Yes | Yes | Yes | **No** | Yes | High risk of bias |
| Cross-sectional study | NA | Dawson [2010] | **No** | **No** | Unclear | Yes | **No** | Yes | Yes | Yes | **No** | High risk of bias |
| Cross-sectional study | 748 | Govender [2010] | Yes | **Unclear** | Unclear | Yes | Yes | **No** | Yes | **No** | Yes | High risk of bias |
| Cross-sectional study | 40 | Middelkoop [2010] | Yes | Yes | Unclear | Yes | Yes | Yes | Unclear | **No** | Yes | Low risk of bias |
| Cross-sectional study | 70 | Cramm [2011] | Yes | Yes | Unclear | **No** | Yes | **No** | Unclear | **No** | Yes | High risk of bias |
| Cross-sectional study | 115 | Lawn [2011] | **No** | **No** | Unclear | Yes | Yes | Yes | Yes | Yes | Yes | High risk of bias |
| Cross-sectional study | 76 | Lawn [2011] | **No** | **No** | Unclear | Yes | Yes | Yes | Yes | Yes | Yes | High risk of bias |
| Cross-sectional study | NA | Kranzer [2012] | Yes | **No** | Unclear | Yes | **No** | Yes | Yes | Yes | **No** | High risk of bias |
| Cross-sectional study | 31 | Lawn [2017] | **No** | **No** | Unclear | Yes | Yes | Yes | Yes | Yes | Yes | High risk of bias |
| Cross-sectional study | 2 | Van Rie [2018] | Yes | Yes | Unclear | Yes | Yes | **No** | Yes | **No** | Yes | High risk of bias |
| Cross-sectional study | 73 | Yates [2018] | **No** | Yes | Unclear | Yes | No | Yes | Unclear | **No** | **No** | High risk of bias |
| Cross-sectional study | 180 | Booi  [2022] | Yes | Yes | Yes | **No** | Yes | **No** | Unclear | **No** | Unclear | High risk of bias |
| **LTBI PREVALENCE** | | | | | | | | | | | | |
| Cohort study | 66 | Wood [2010] | Yes | **Yes** | Unclear | **No** | NA | Yes | Unclear | Yes | NA | Low risk of bias |
| Cross-sectional study | 23 | Du Preez [2011] | **No** | **No** | Unclear | Yes | Unclear | Yes | Yes | **No** | Unclear | High risk of bias |
| Cross-sectional study | 99 | Middelkoop [2014] | Yes | Yes | Unclear | Yes | Unclear | Yes | Yes | Yes | Yes | Low risk of bias |
| Cross-sectional study | 170 | Ncayiyana [2015] | Yes | Yes | Unclear | Yes | Yes | Yes | Yes | Yes | Unclear | Low risk of bias |
| Cross-sectional study | 111 | Bunyasi [2019] | **No** | **No** | Yes | Yes | Unclear | Yes | Yes | Yes | Unclear | High risk of bias |
| **TB INCIDENCE** | | | | | | | | | | | | |
| Cross-sectional study | 104 | Wood [2010] | Yes | **No** | Unclear | Yes | Unclear | Yes | Unclear | Yes | Unclear | High risk of bias |
| Cohort study | 810 | Gupta [2012] | **No** | **No** | Unclear | Yes | Unclear | Yes | **No** | Yes | Yes | High risk of bias |
| Cohort study | 4627 | Naidoo [2014] | **No** | **No** | Unclear | Yes | **No** | **No** | Yes | Yes | Yes | High risk of bias |
| Cohort study | 91 | Martinez [2017] | Yes | Yes | Unclear | Yes | Unclear | Yes | Yes | Yes | Yes | Low risk of bias |
| Cohort study | 64 | Ilunga [2020] | Yes | Yes | Yes | Yes | Yes | **No** | Unclear | **No** | Unclear | High risk of bias |
| **LTBI INCIDENCE** | | | | | | | | | | | | |
| Cross-sectional study | 99 | Middelkoop [2014] | Yes | Yes | Unclear | Yes | Unclear | Yes | Yes | Yes | Yes | Low risk of bias |
| Cohort study | 91 | Martinez [2017] | Yes | Yes | Unclear | Yes | Unclear | Yes | Yes | Yes | Yes | Low risk of bias |

**Abbreviations:** *TB – Tuberculosis; LTBI = Latent Tuberculosis; NA = Not applicable.*

**Legend:** **Because of the importance of sample frame (item 1), sampling approach (item 2) and diagnostic methods (item 6) for determining prevalence and incidence in a target population, studies receiving a ‘No” response for any of these items were labelled as ‘high risk-of-bias’.*

**Table B in S4 Table**. Justifications for overall appraisal

| **Study**  **ID** | **Author**  **[Year]** | **Justification if ‘No’ or ‘Unclear’ was selected for any of the assessment items** | **Justification for overall appraisal** |
| --- | --- | --- | --- |
| **TB PREVALENCE** | | | |
| 80 | Cox [2010] | - 1. Study conducted in 2 primary care clinic for adults suspected to have pulmonary TB (may be more likely to be symptomatic and have TB than the source population); - 2. Participant recruitment through convenience sampling and not population-based sampling which is more likely to yield a representative study sample; - 3. No sample size for TB prevalence calculated (primary outcome of this study was not to obtain a TB prevalence estimate); - 8. The percentage of patients with TB (TB prevalence) was not reported with a confidence interval (primary outcome of this study was not to obtain a TB prevalence estimate) | Despite the use of an appropriate diagnostic tool, the sample (clinic attendees with symptoms of TB) may not be representative of our target population. This study aims to assess the proportion of TB cases that are drug-resistant, which explains why the study sample did not use population-based sampling |
| NA | Dawson [2010] | - 1. Study participants were recruited from an ART service and were HIV positive which is not representative of the source population (overestimation of TB prevalence); - 2. Use of a convenience sample (HIV pos. patients attending an ART-clinic are HIV pos. and possibly more health-conscious than the source population) which may endanger generalizability; - 3. No sample size calculation was conducted (the primary outcome of this study was not to obtain a TB prevalence estimate); - 5. Overrepresentation of women (73% of the study sample) potentially impairs the generalizability of results; - 9. The potential impact of the 74% female study sample on the TB prevalence was not discussed | This study should be excluded because a) the sample population is not representative of the source population (73% women, all HIV pos.), and b) the small sample size (n=235). |
| 748 | Govender [2010] | - 2. It is unclear how survey participants (dwellings) were selected; - 3. No sample size calculation was performed but the sample size was very small (n=370 for shack inhabitants); - 6. TB status was obtained through an interview questionnaire (highly subjective, voluntary disclosure of TB status most likely underestimates true TB count); - 8. Percentage of individuals with TB reported as % without confidence interval | This study should be excluded, mainly because of the diagnostic tool that was used to diagnose TB (self-report) but also because of the small sample size. |
| 40 | Middelkoop [2010] | - 3. No sample size calculation was conducted by the authors but the sample was randomly chosen from a large community (?); - 5. 90% of approached residents agreed to participate in the study, which is considered a sufficiently high response rate; - 7. The outcome ascertainment process is described in detail, and although there is no information on the level of training of those who collected the data, collection of sputum samples can be considered sufficiently objective to guarantee standardized measurement of TB; - 8. Confidence intervals for the TB prevalence (%) are not provided | Despite the missing confidence interval for the TB prevalence estimate and the missing sample size calculation, the well-described methods (including standardized case definitions, rigorous case ascertainment, population-based sampling), high response rate and representative population warrant inclusion of this study. |
| 70 | Cramm [2011] | - 3. No sample size calculation conducted by the authors; - 4. Demographic characteristics of the population are not described in detail (e.g. sex/gender, age-distribution, co-morbidities such as HIV); - 5. A 97.9% response rate can be considered sufficiently high; - 6. Use of self-reported TB on a household-level is not an objective measurement of TB prevalence; - 7. No information available on the level of training of the researchers who conducted the household surveys and how standardized/comparable the data collection was; - 8. Unclear what the denominator was (977 vs 1020), TB prevalence had to be hand-calculated, TB case numbers not stated (had to be derived from a percentage), no confidence interval reported | Exclusion of this study mainly because of the use of a highly subjective, non-standardized TB diagnostic tool which may underestimate the true TB burden |
| 115 | Lawn [2011] | - 1. Study participants were recruited from an ART service and were HIV positive which is not representative of the source population (overestimation of TB prevalence); - 2. Use of a convenience sample (HIV pos. patients attending an ART-clinic are HIV pos. and possibly more health-conscious than the source population) which may endanger generalizability; - 3. No sample size calculation was conducted (the primary outcome of this study was not to obtain a TB prevalence estimate); - 7. Collection of sputum samples: although it is not described who took the sputum samples (level of training, technique), this can be considered a standardized procedure and is thus sufficiently well described | This study should be excluded because a) the study sample is not representative of the source population, b) no sample size calculation was conducted, and the sample size is relatively small |
| 76 | Lawn [2011] | - 1. Study was exclusively conducted in HIV pos. individuals which is not representative of the source population; - 2. Use of a convenience sample (HIV pos. patients attending an ART-clinic are HIV pos. and possibly more health-conscious than the source population) which may endanger generalizability; - 3. No sample size calculation was conducted (the primary outcome of this study was not to obtain a TB prevalence estimate); | This study should be excluded because the study sample is not representative of the source population. |
| NA | Kranzer [2012] | - 2. Use of convenience sampling (mobile testing service located near shopping centers, taxi ranks, stations and the road side); - 3. No sample size calculation was performed; - 5. Men were heavily underrepresented in the study sample (35.9%), - 9. Impact of poor response rate of men on TB prevalence was not discussed | This study should not be included (use of a convenience sample, low uptake of TB screening services, heavily female-dominated study sample). |
| 31 | Lawn [2017] | - 1. Enrollment of hospitalized, HIV pos. individuals only (patients may be sicker than the general population and more likely to have co-morbidities, including TB and/or other conditions that increase their TB risk); - 2. Use of a convenience sample, no random population-based sampling technique employed; - 3. No sample size calculation performed (TB prevalence not the primary study outcome) | This study should be excluded because it was performed in a population that is not representative of the source population (HIV. pos, hospitalized individuals) and the sample size was small (n=427). |
| 2 | Van Rie [2018] | - 3. No sample size calculation was performed (it should be noted that this is a purely descriptive study and no hypothesis was tested); - 5. Despite 39% refusal rate, the geographical distribution of the sample was reported as balanced, with a balanced age range of participants and 55% female participants; - 6. NAAT only performed in individuals with presumptive TB based on TB symptom screening (subjective non-standardized TB diagnostic tool); - 8. Percentage of participants with TB reported as percentage without confidence interval | This study should be reported because of the use of an objective TB diagnostic tool (bacterial culture) used in a standardized way, use of a population-based random sampling framework and relatively balanced sample characteristics. |
| 73 | Yates [2018] | - 1. The study population is an impoverished population defined as having a household wealth index of "low" and "very low" (low SES/poverty). Because the calculation of this index is insufficiently defined in the paper, the “exposed” study population may not be representative of the target population; - 3. No sample size calculation was conducted; - 5. Enrollment rate of 78% but underrepresentation of men (make up only<40% of the sample which may bias the overall TB prevalence downward); - 7. TB culture methods are not described in detail (e.g. sputum induction, level of training of staff that took the sample, culture methods); - 8. Percentage of participants who were TB pos. given without a confidence interval (TB prevalence was not the primary outcome of this study); - 9. The study did not discuss the potential impact of underrepresenting men in the study sample on TB prevalence | This study should be excluded because a) the study population is most likely not entirely representative of the target population, and b) the underrepresentation of men in the study sample may bias the TB prevalence estimate. |
| 180 | Booi [2022] | - 3. Sample size calculation not conducted, but the sample size is large and includes the entire community of the township so no sample size calculation is required; - 4. No demographic information provided beyond the fact that the survey was conducted in a township population; - 6. TB case ascertainment done using voluntary TB-disclosure (household survey) on a household level; - 7. Unclear who conducted the household survey or what their level of training was; - 8. TB case numbers given but no percentage or confidence interval (TB prevalence was also not the primary study outcome); - 9. No information on the response rate provided | This study should be excluded because of the diagnostic tool used to ascertain TB cases (subjective self-report) and the lack of any baseline information on study participants that would allow us to estimate the generalizability of study results. |
| **LTBI PREVALENCE** | | | |
| 66 | Wood [2010] | - 2. Use of a convenience sample (adults were recruited from a single primary health clinic); however, the clinic is reported to be the only health care clinic within the study community and it can be assumed that all TB cases within the community were registered there; - 3. No sample size calculation was conducted (TB prevalence was not the primary outcome of this study); - 4. Study participants' baseline characteristics were not described in detail using a table or a descriptive summary (e.g. sex/gender, co-morbidities, age, etc); - 5. Not applicable. because the TB data was extracted from a community TB register based on TB case notifications collected by the primary health clinic (no response rate bias); - 7. Unclear because we do not know who conducted TB tests (level of training) and what technique was used; - 9. No applicable because the response rate does not apply in this study | Despite the lack of a study participant characteristics table and information on the technique used to culture TB, this study should be included because TB data was collected in a clinic setting (trained staff), the large sample size and long follow-up period (4 years). |
| 23 | Du Preez [2011] | - 1. Inclusion of children with household contact to a TB pos. adult only (not a representative sample of the source population); - 2. It is unclear how children were sampled and selected into the study (it is only mentioned that included children's TB pos. household contact must have started anti-TB treatment in the preceding 3 months, which sounds like a convenience sample since only treated adults were included); - 3. No sample size calculation was conducted (LTBI prevalence is not the primary outcome); - 5. Unclear, because the response rate is not given; 8. Percentage of children with TB reported without confidence intervals; - 9. No response rate provided | This study should be excluded because of its unrepresentative sample of the source population (children of TB pos. adults that were treated for TB only) and extremely small sample size (n=196). |
| 99 | Middelkoop [2014] | - 2. No random sampling framework was employed but in participating schools all students were included; - 3. No sample size calculation was performed (LTBI prevalence/incidence was not the primary outcome of this study); - 5. No information given on number of participants approached vs number of participants that consented/enrolled into the study (no refusal rate given); - 9. Unclear what the response rate was and/or how it was managed, however, the distribution of participants looks balanced (50% female, age range covers school-age children) | Despite the lack of sample size calculation and study participation refusal rate, the baseline characteristics of included participants look balanced, a standard test for the diagnosis of LTBI was used. Thus, this study should be included. |
| 170 | Ncayiyana [2015] | - 3. No sample size calculation was performed; - 9. Out of 1620 randomly selected households, 1581 (97.6%) could be contacted and 1230 (77.8%) agreed to participate and it is not explained what the reasons for refusal. Further, 144/626 participants refused the TST for which the reasons are not given either. This is briefly discussed in the discussion section but no attempts are made to correct this. | This study should be included because of its overall rigorous methods. |
| 111 | Bunyasi [2019] | - 1. Study participants were recruited from state-funded high schools near Cape Town and stratified into quintiles according to SES. Schools in quintiles 1-4 were compared against schools in quintile 5 and quintiles 1-4 considered exposed (= low SES) but it is questionable how well school children in quintiles 1-4 fit into our exposure definition; - 5. No information provided on the number of participants approached vs the number of participants that consented/enrolled (no refusal rate given); - 9. No response rate given | This study should be excluded despite its overall good quality assessment, mainly because it is unclear if the exposure definition in this study fits the exposure definition for our study (see point 1.). |
| **TB INCIDENCE** | | | |
| 104 | Wood [2010] | - 2. Use of a convenience sample (HIV voluntary counseling/testing centre, school attendees) which may overrepresent health-conscious, educated children and adults, exclusion of HIV negative individuals which does not reflect the target population; - 3. No sample size calculation conducted; - 5. No information provided on response bias (no details given on number approached vs number responded/enrolled); - 7. No information provided on who conducted the TST on study participants, who interpreted it and how diverging interpretations were handled; - 10. No information on response rate provided | This study should be excluded because of uncertainty around the representativeness of the study sample compared to the source population we are interested in. |
| 810 | Gupta [2012] | - 1. Enrollment of HIV. pos individuals only; - 2. Study participants were recruited from an ART service (only attended by HIV pos. individuals), which does not adequately reflect the source population (in which not everyone is HIV pos.); - 3. No sample size calculation performed; - 5. Individuals who deferred ART (119/2000) were excluded from analysis and may differ from participants who did not defer ART (SES for example may be associated with both TB status and ability to initiate ART --> bias towards the null and underestimation of true TB incidence); - 7. TB was diagnosed based on a variety of different diagnostic tools that differed between patients; - 9. Among those with TB, 19.6% were lost to FU, among those without TB 21% were lost to FU. Although these numbers are not insignificant, they are almost identical between those with and without a TB diagnosis (differential loss to FU unlikely) | The main reason why this study should be excluded is its focus on HIV positive individuals, which most likely overestimates the true TB incidence in the source population. Further, only 73% of TB cases were culture-confirmed and diagnostic methods were not uniform. |
| 4627 | Naidoo [2014] | - 1. Only HIV pos individuals initiating HAART in this particular program were enrolled and 2/3 of the population were women; - 2. Participants were not enrolled into the study using random sampling (use of a convenience sample); - 3. No sample size calculation conducted; - 5. Only 1/3 of the population were male; 6. Only individuals with a pos. TB symptom screening results were referred to TB smear microscopy | Exclusion of this study mainly because of the choice of diagnostic tool (not WHO-approved), because the sample is not representative of the source population (HIV pos undergoing HAART), and because of the poor sampling (convenience sample) |
| 91 | Martinez [2017] | - 3. No sample size calculation provided although LTBI incidence is the primary outcome of this study; - 5. No information given on the number of participants approached vs the number of participants enrolled/consented | This study should be included because of its overall methodological rigor, valid diagnostic tools and representative study sample. |
| 64 | Ilunga [2020] | - 2. Sampling methods are not reported, however, AitaHealth is a community-wide implemented TB screening program that covers a large amount of households in the source population and can be expected to yield a balanced sample; - 3. No sample size calculation conducted but the entire population was included in the TB screening program and the sample size is large (n=184,351); - 6. Use of self-report to diagnose TB (reporting bias, underestimation of TB incidence); - 7. No information provided on how information on a diagnosis of TB was collected (e.g. questionnaire, training level of interviewer, TB case definition); - 8. The percentage of individuals with TB was reported without confidence interval; - 9. Response rate not reported | This study should be excluded because of the highly subjective diagnostic tool used to diagnose TB (self-report). |
| **LTBI INCIDENCE** | | | |
| 99 | Middelkoop [2014] | - 2. No random sampling framework was employed but in participating schools all students were included; - 3. No sample size calculation was performed (LTBI prevalence/incidence was not the primary outcome of this study); - 5. No information given on number of participants approached vs number of participants that consented/enrolled into the study (no refusal rate given); - 9. Unclear what the response rate was and/or how it was managed, however, the distribution of participants looks balanced (50% female, age range covers school-age children) | Despite the lack of sample size calculation and study participation refusal rate, the baseline characteristics of included participants look balanced, a standard test for the diagnosis of LTBI was used. Thus, this study should be included. |
| 91 | Martinez [2017] | - 3. No sample size calculation provided although LTBI incidence is the primary outcome of this study; - 5. No information given on the number of participants approached vs the number of participants enrolled/consented | This study should be included because of its overall methodological rigor, valid diagnostic tools and representative study sample. |
