# Supplementary material for: Burden of tuberculosis in underserved populations in South Africa: A systematic review and meta-analysis
Source: PLOS Glob Public Health. 2024 Oct 3;4(10):e0003753. doi: 10.1371/journal.pgph.0003753 (PMC11449336; doi:10.1371/journal.pgph.0003753)
Supplement: S5 Table — (DOCX) [file pgph.0003753.s006.docx]

## **S5 Table**. Egger Tests: P-values

| **Outcome** | **HIV status** | **p-value** |
| --- | --- | --- |
| TB prevalence | People living with HIV | 0.8904 |
| TB prevalence | People living with and without HIV | 0.4617 |
| LTBI prevalence | People living without HIV | 0.8645 |
