# Supplementary material for: Burden of tuberculosis in underserved populations in South Africa: A systematic review and meta-analysis
Source: PLOS Glob Public Health. 2024 Oct 3;4(10):e0003753. doi: 10.1371/journal.pgph.0003753 (PMC11449336; doi:10.1371/journal.pgph.0003753)
Supplement: S6 Table — (DOCX) [file pgph.0003753.s007.docx]

## **S6 Table**. Pooled Prevalence before and after Sensitivity Analysis

| **Outcome** | **HIV status** | **Pooled prevalence (before sensitivity analysis)** | **Pooled prevalence (after sensitivity analysis)** |
| --- | --- | --- | --- |
| TB prevalence | People living with HIV | 15.43 % (95 % CI 7.7 - 25.3 %) | 22.65 % (95 % CI 15.8 - 30.4 %) |
| TB prevalence | People living without HIV | 2.7 % (95% CI 0.1 - 8.5 %) | Unchanged |
| TB prevalence | People living with and without HIV | 7.92 % (95% CI 0.4 - 23 %) | 2.91 % (95 % CI 1.5 - 4.8 %) |
| LTBI prevalence | People living with HIV | 33.03 % (95% CI 22.6-44.4%) | Unchanged |
| LTBI prevalence | People living without HIV | 43.42 % (95 % CI 39.5 - 47.3 %) | 44.78% (95 % CI 42.5 - 47 %) |
| LTBI prevalence | People living with and without HIV | 54.6 % (95% CI 53.3 - 55.9 %) | Unchanged |
