# Supplementary material for: Burden of tuberculosis in underserved populations in South Africa: A systematic review and meta-analysis
Source: PLOS Glob Public Health. 2024 Oct 3;4(10):e0003753. doi: 10.1371/journal.pgph.0003753 (PMC11449336; doi:10.1371/journal.pgph.0003753)
Supplement: S1 Data — (DOCX) [file pgph.0003753.s008.docx]

## **S1 Data**. Sensitivity Analysis

### TB Prevalence

#### People with HIV

**Leave-one-out analysis, Baujat plots & Diagnostic tests**


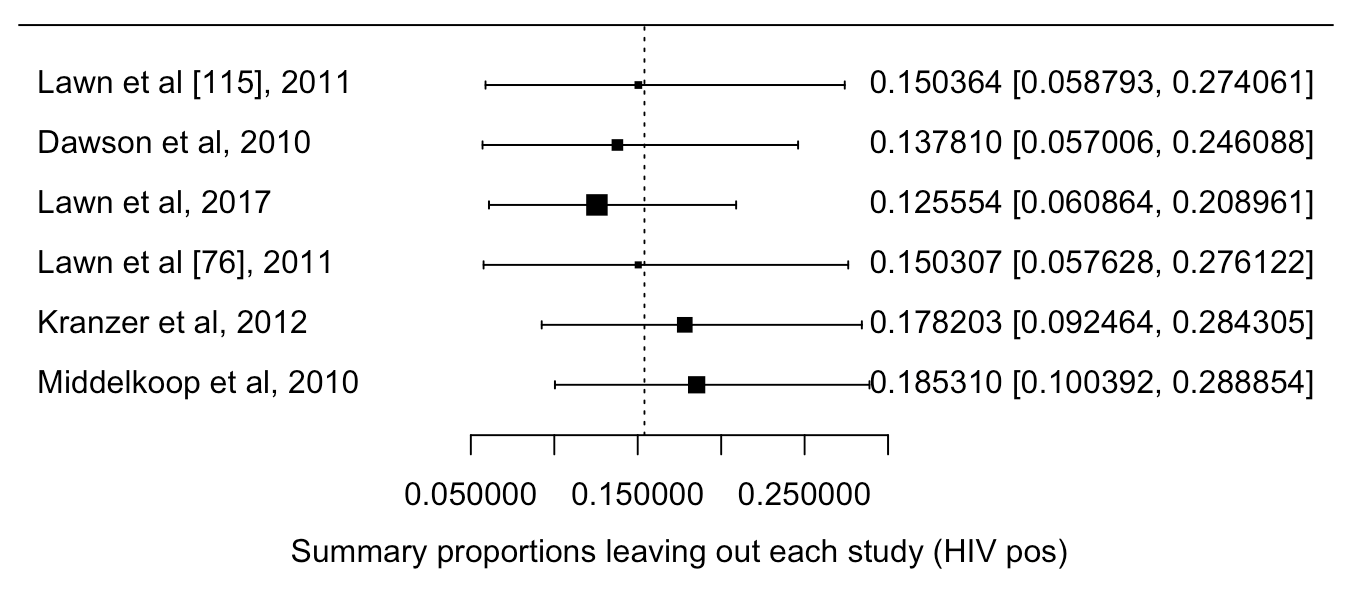


**Interpretation**: Study 3, 5, and 6 have most impact on the summary proportion


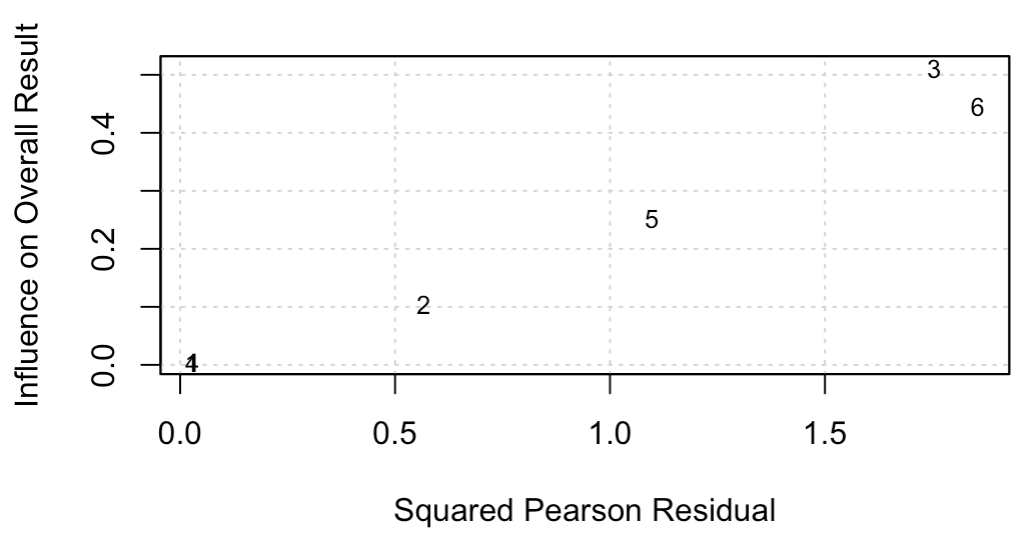


**Interpretation**: Study 3, and 6 (5 to a lesser degree) have most impact on the summary proportion


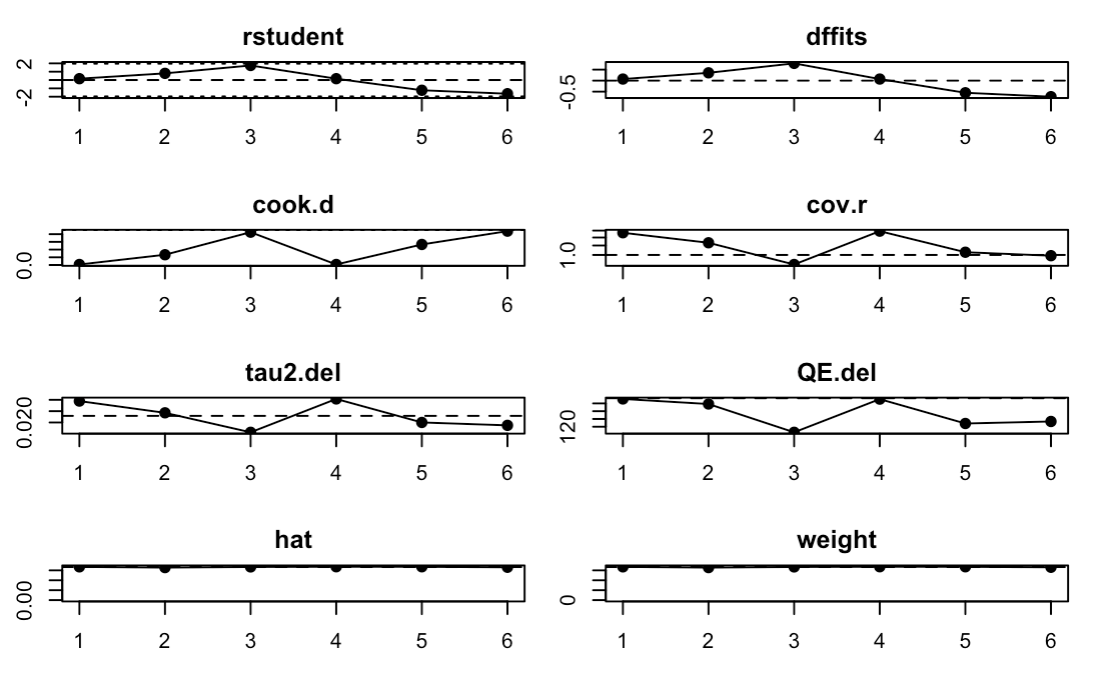


**Interpretation**: No study has a significant impact on the summary proportion (significant studies highlighted in red)

**Conclusion**: None of the included studies was identified as a statistically significant outlier. However, all studies apart from studies 5 (*Kranzer et al* (2012)) and 6 (*Middelkoop et al* (2010)) were exclusively conducted in PLWH at HIV treatment clinics or testing centers situated in townships. Studies 5 and 6 were conducted in people living with and without HIV of low socio-economic status and situated in a township, respectively. Because we expect the population’s HIV status and associated co-morbidities, as well as differences in study procedures and study settings (i.e. hospital patients tend to be more comorbid than patients recruited in outpatient settings, patients are under closer supervision in hospital settings) to be major determinants of heterogeneity, these studies were excluded.

#### People with and without HIV

**Leave-one-out analysis, Baujat plots & Diagnostic tests**


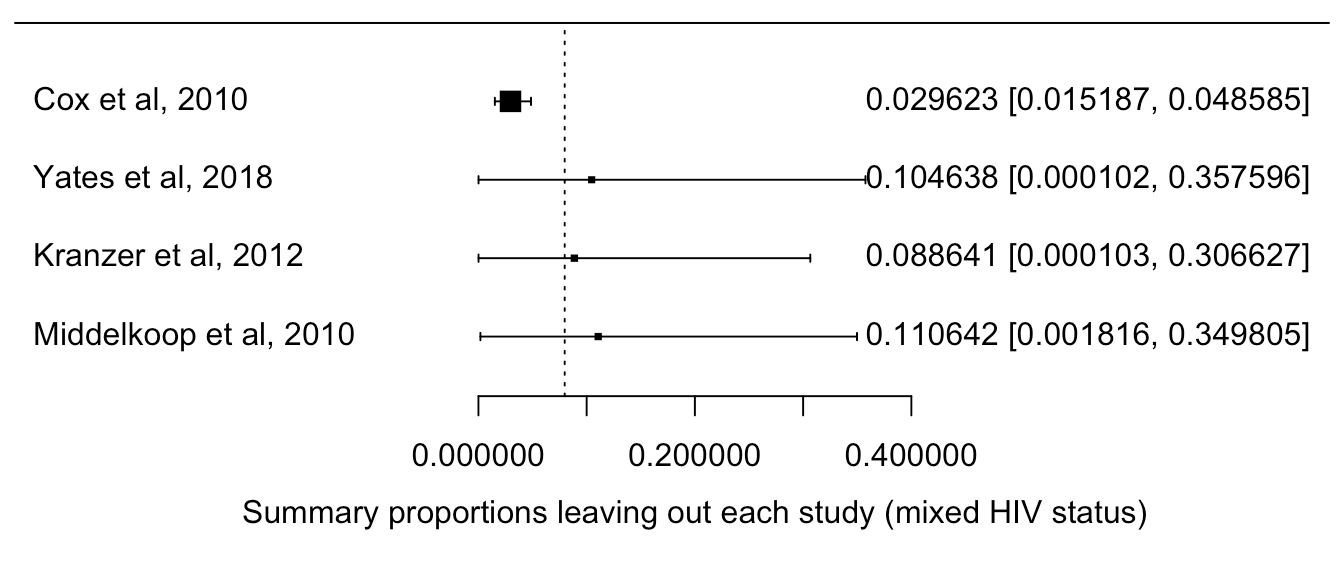


**Interpretation**: Study 1 has most impact on the summary proportion


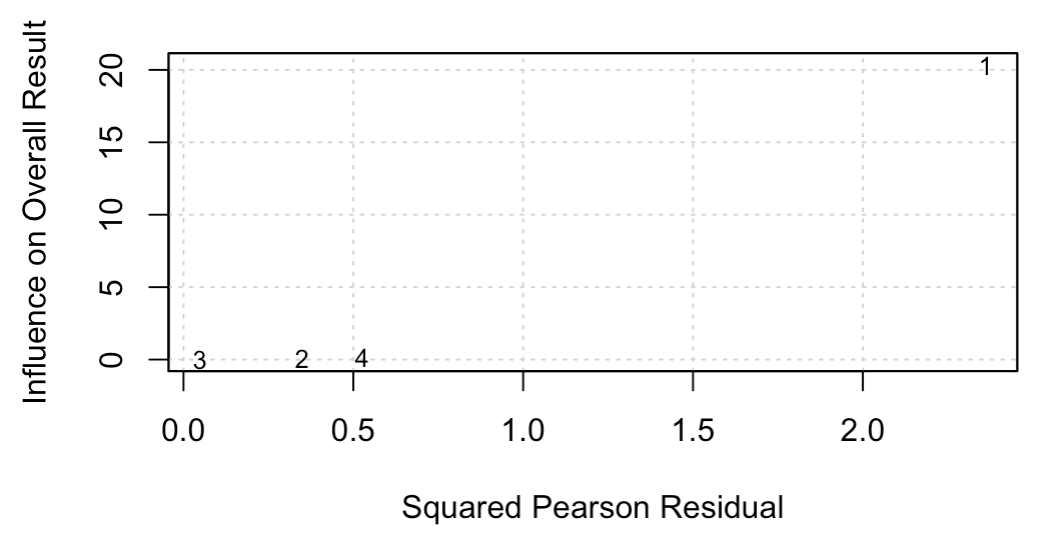


**Interpretation**: Study 1 has most impact on the summary proportion


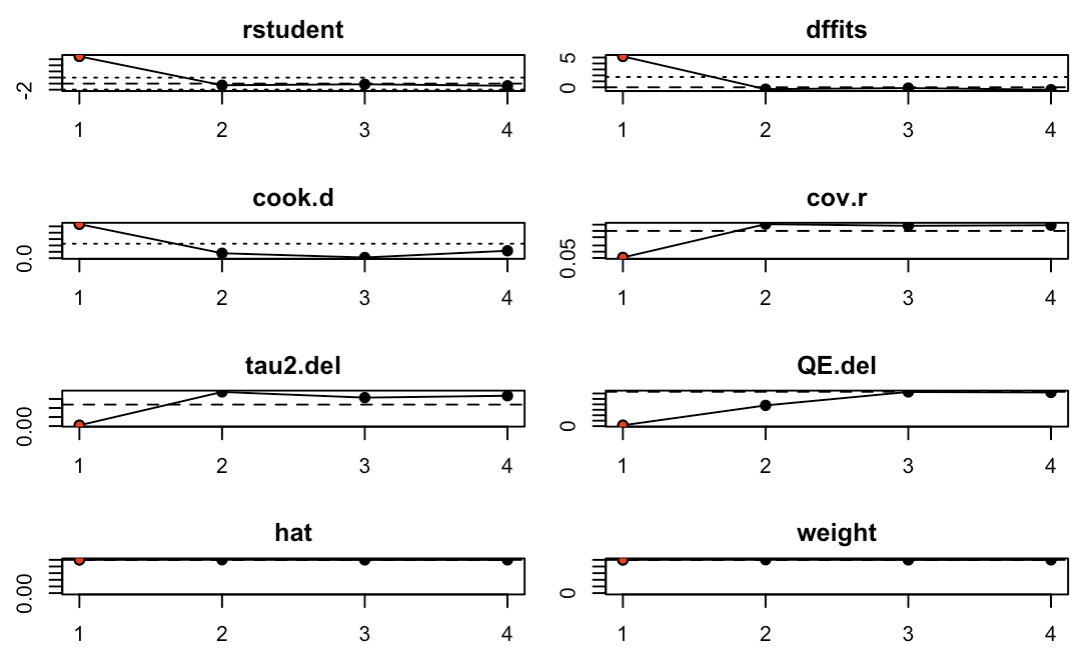

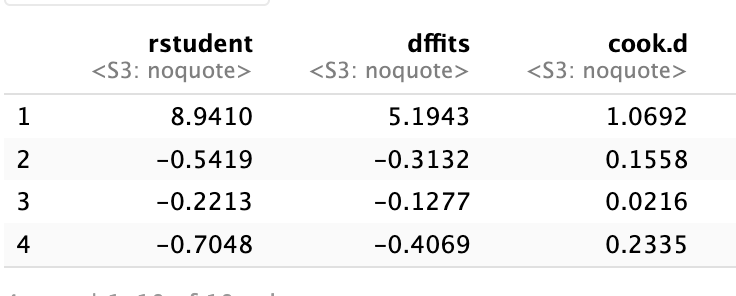


**Interpretation**: Study 1 has a significant impact on the summary proportion, with a Cook’s distance of 1.0692, as highlighted in red. A point for which Cook’s distance is >1 is almost universally considered as influential. Further, using the 4/n significance threshold (n = the total number of data points), study 1 is also considered as influential. Lastly, visual inspection also shows that the value of study 1 is substantially larger than the rest[2].

**Conclusion**: Study 1 is a statistically significant outlier. Thus, the study was excluded.

### LTBI Prevalence

#### People without HIV

**Leave-one-out analysis, Baujat plots & Diagnostic tests**

###
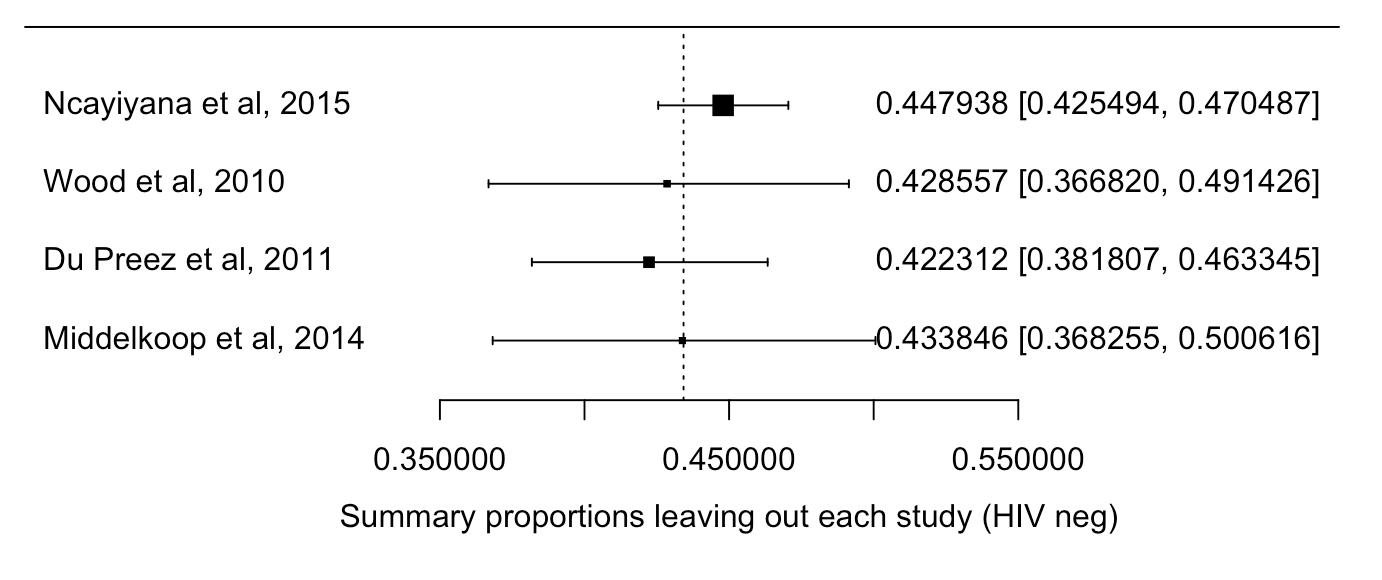


**Interpretation**: Study 1 has a slight impact on the summary proportion
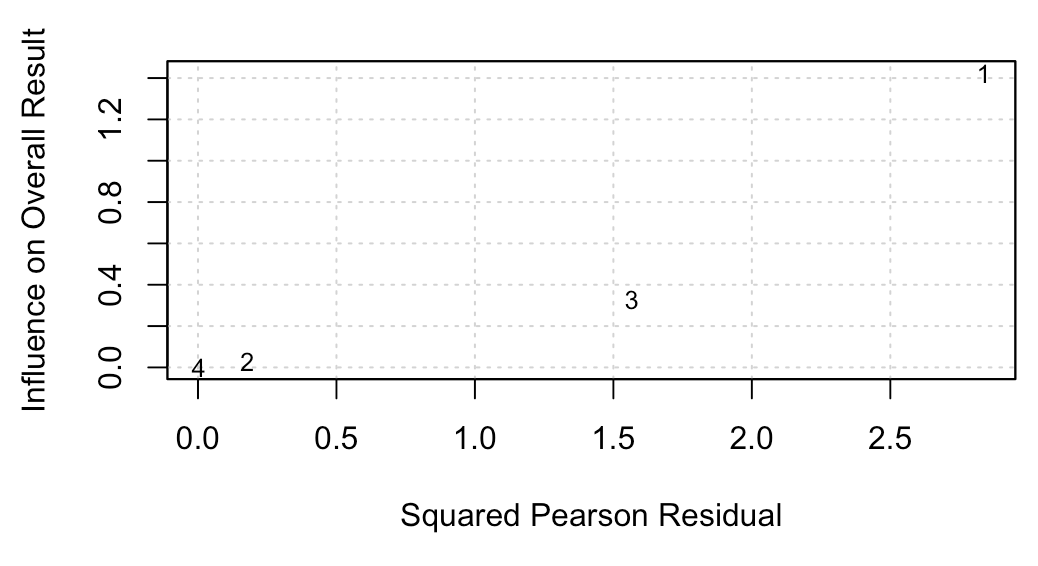


**Interpretation**: Study 1 has a significant impact on the summary proportion
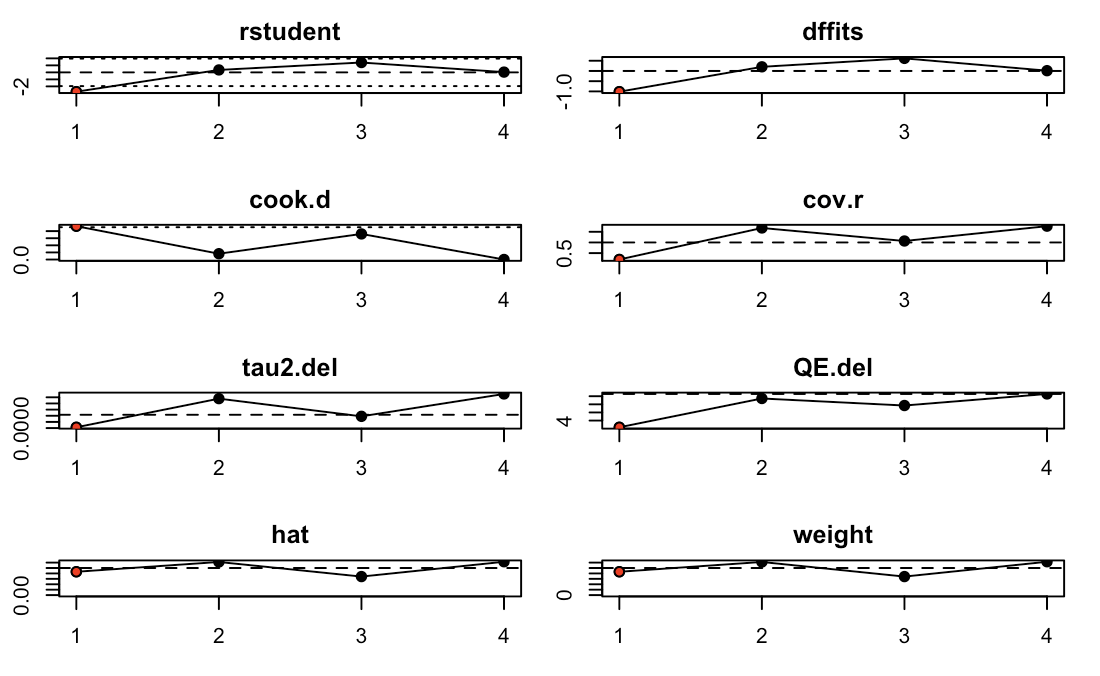

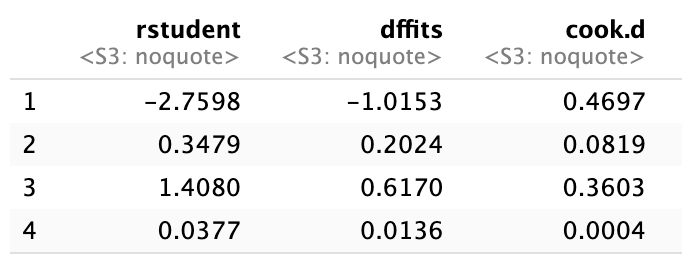


**Interpretation**: Study 1 has a significant impact on the summary proportion with a Cook’s distance of 0.4697, as highlighted in red. While the Cook’s distance is <1 and does not meet the 4/n significance threshold (n = the total number of data points), visual inspection shows that the value of study is substantially larger than the rest.

**Conclusion**: Study 1 is a statistically significant outlier. Thus, the study was excluded.
