# Supplementary material for: Burden of tuberculosis in underserved populations in South Africa: A systematic review and meta-analysis
Source: PLOS Glob Public Health. 2024 Oct 3;4(10):e0003753. doi: 10.1371/journal.pgph.0003753 (PMC11449336; doi:10.1371/journal.pgph.0003753)
Supplement: S2 Data — (DOCX) [file pgph.0003753.s009.docx]

## **S2 Data**. Prevalence Ratios

All formulas were adapted from *Boston University School of Public Health*’s online course on Measures of Disease Frequency and Association [3]. Formulas were designed with *mathcha* [4].

### Data Sources

| **Source Estimate (Year)** | | | **Value** | **Rationale** |
| --- | --- | --- | --- | --- |
| **National TB Prevalence Survey 2018^[5]^** | National TB prevalence (2018) | | **Bacteriologically confirmed TB (all ages)**: **737 (95% CI 580-890) → 0.74%**  Reference: Page 17 | Most accurate and up to date national prevalence estimate |
|  |  |  |  |  |
| ***Rein et al:***  **The Global Burden of Latent Tuberculosis Infection^[6]^** | National LTBI prevalence (2014) | | **LTBI prevalence (all ages):** 17,200,000 / 54,000,000 = 0.3185 (**31.85%**)  **LTBI prevalence (<15 LTBI):** 2,200,000 / 16,200,000 = 0.1358 (**13.58%**) | Only available national LTBI prevalence estimate in pediatric populations |
|  |  |  |  |  |
| ***Mid-year population estimates 2014^[7]^*** | Population size South Africa (2014) | | **Entire population**: 54,000,000  **<15 years (30%)**:  16,200,000 | Calculation of national LTBI prevalence based on the TB case number extracted from *Rein et al* (numerator) and the mid-year population size of 2014 (denominator) |

### Calculations

| **TB prevalence** | **Formula** | **Calculation** |
| --- | --- | --- |
| **People living with HIV**: 0.2265 [95% CI 0.1575 - 0.3039]  **People living without HIV**: 0.0279 [95% CI 0.0007 - 0.0846]  **People living with and without HIV**: 0.0291 [95% CI 0.0147 - 0.0481]  **Overall population, 2018**: 0.00737 (95% CI: 0.0058 - 0.0089) | 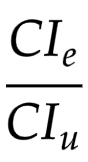  C_u_ = Prevalence in the exposed (underserved populations)  C_e_ = Prevalence in the unexposed (national estimate) | Cl_e_ (People living with HIV)/Cl_u_: 0.2265 / 0.00737 = **30.7327**  Cl_e_ (People living without HIV)/Cl_u_: 0.0279 / 0.00737 = **3.7856**  Cl_e_ (People living with and without HIV)/Cl_u_: 0.0291 / 0.00737 = **3.9484** |
|  | | |
| **LTBI prevalence** | **Formula** | **Calculation** |

| **People living with HIV**: 0.3302 [95% CI 0.2255 - 0.4442] - single-study estimate  **People living without HIV**: 0.4478 [95% CI 0.4254 - 0.4704]  **People living with and without HIV**: 0.5460 [95% CI 0.5332 - 0.5587] - single-study estimate  **LTBI prevalence (Overall population <15 years, 2014)**: 0.1358 (used for the HIV negative and mixed HIV status subgroups because contributing studies were conducted in pediatric/adolescent populations)  **LTBI prevalence (Overall population, age-standardized, 2014)**: 0.3185 (used for the HIV positive status subgroups because the contributing study was conducted in a population of all ages) | 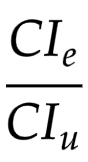  C_u_ = Prevalence in the exposed (underserved populations)  C_e_ = Prevalence in the unexposed (national estimate) | Cle (People living with HIV)/Clu: 0.3302 / 0.3185 = **1.0367**  Cle (People living without HIV)/Clu: 0.4478 / 0.1358 = **3.2975**  Cle (People living with and without HIV)/Clu: 0.5460 / 0.1358 = **4.0206** |
| --- | --- | --- |

### Prevalence Ratios - Overview

| **Outcome** | **People living with HIV** | **People living without HIV** | **People living with and without HIV** |
| --- | --- | --- | --- |
| **TB Prevalence** | PR = 30.7327 | PR = 3.7856 | PR = 3.9484 |
| **LTBI Prevalence** | PR = 1.0367  → Single-study estimate with small sample size | PR = 3.2975 | PR = 4.0206  → Single-study estimate |

[3] Boston University. (2021). *PH717 - Module 3 - Measuring Frequency and Association*. Retrieved Jan 21, 2024 from <https://sphweb.bumc.bu.edu/otlt/MPH-Modules/PH717-QuantCore/PH717-Module3-Frequency-Association/index.html>

[4] Mathcha. (2022). *Mathcha Editor*. Mathcha. Retrieved Jan 21, 2024 from <https://www.mathcha.io>

[5] Council, S. A. M. R. (2018). *The First national TB Prevalence Survey* (Report). N. D. o. Health. <https://www.samrc.ac.za/media-release/first-south-african-national-tb-prevalence-survey-gives-clearer-picture-epidemic>

[6] Houben, R. M., & Dodd, P. J. (2016). The Global Burden of Latent Tuberculosis Infection: A Re-estimation Using Mathematical Modelling. *PLoS Med*, *13*(10), e1002152. <https://doi.org/10.1371/journal.pmed.1002152>

[7] Statistics South Africa. (2014). *Mid-year population estimates 2014*. <https://www.statssa.gov.za/publications/P0302/P03022014.pdf>
