# Supplementary material for: Burden of tuberculosis in underserved populations in South Africa: A systematic review and meta-analysis
Source: PLOS Glob Public Health. 2024 Oct 3;4(10):e0003753. doi: 10.1371/journal.pgph.0003753 (PMC11449336; doi:10.1371/journal.pgph.0003753)
Supplement: S1 Fig — Abbreviations: HIV = Human Immunodeficiency Virus. *The ‘People living with and without HIV’ group includes studies for which outcomes were not stratified by HIV status. (DOCX) [file pgph.0003753.s011.docx]

## **S1 Fig**. Meta-analysis of TB Prevalence (Random-effects Model)

*
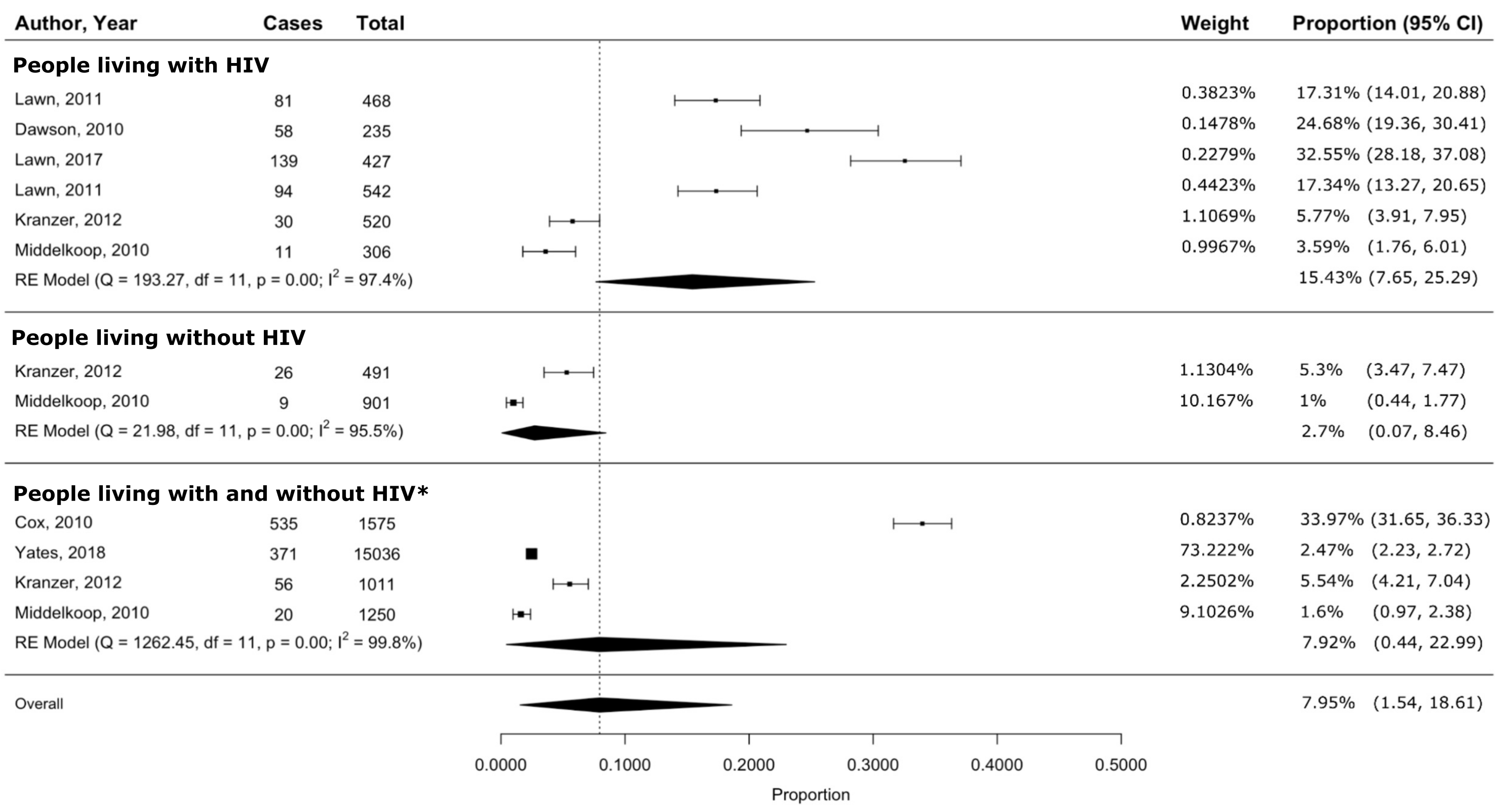
*

***S1 Fig****. Pooled active TB disease prevalence among underserved populations in South Africa, stratified by HIV status.*

***Abbreviations****: HIV = Human Immunodeficiency Virus.*

***Legend****: *The ‘People living with and without HIV’ group includes studies for which outcomes were not stratified by HIV status.*
