# Supplementary material for: Burden of tuberculosis in underserved populations in South Africa: A systematic review and meta-analysis
Source: PLOS Glob Public Health. 2024 Oct 3;4(10):e0003753. doi: 10.1371/journal.pgph.0003753 (PMC11449336; doi:10.1371/journal.pgph.0003753)
Supplement: S3 Fig — Abbreviations: TB = Tuberculosis; HIV = Human Immunodeficiency Virus. (DOCX) [file pgph.0003753.s013.docx]

## **S3 Fig**. Funnel plot: TB Prevalence (People living without HIV)


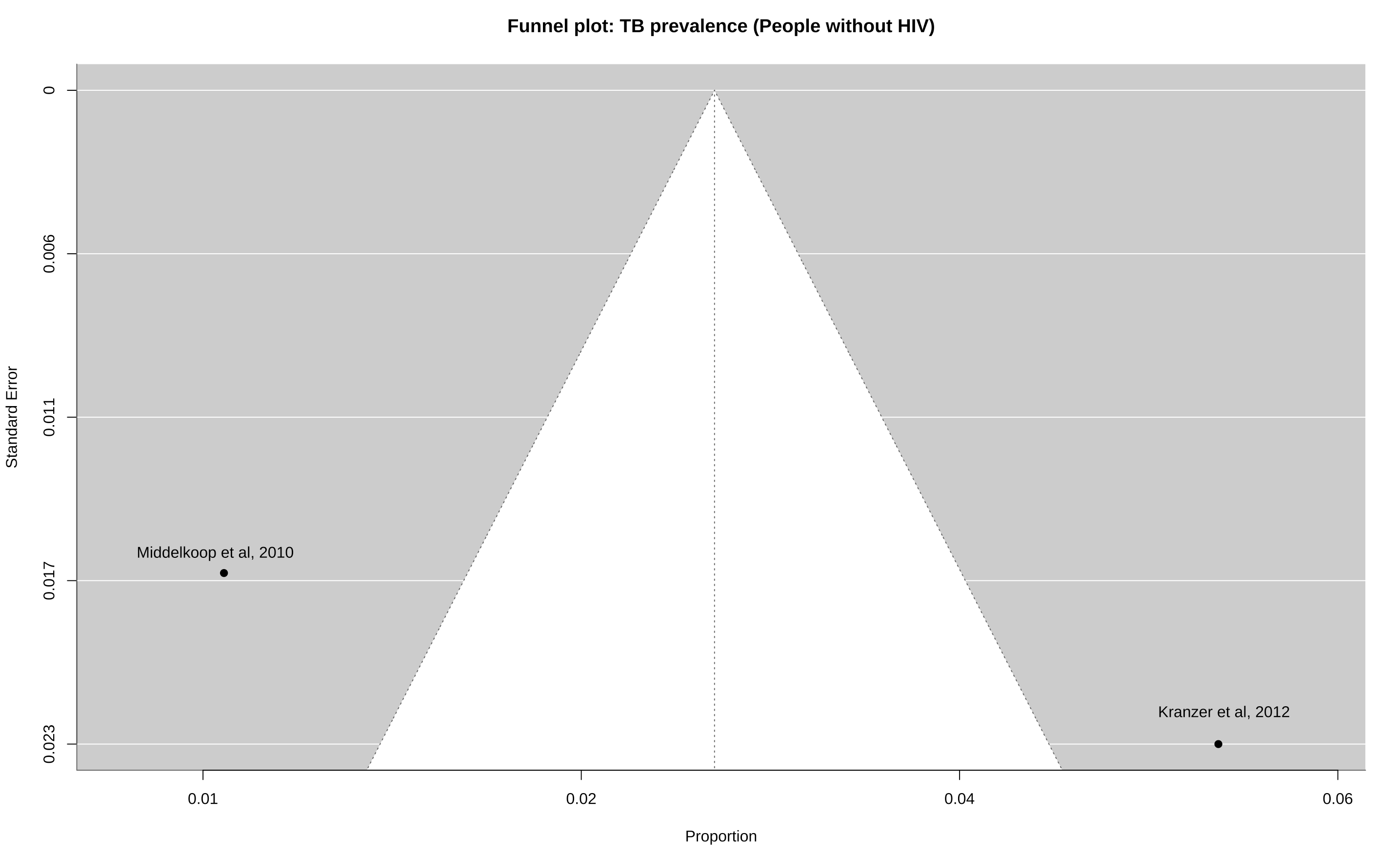


***Fig S3****. Funnel plot: Pooled active TB disease prevalence among underserved populations in South Africa (‘People living without HIV’ subgroup).*

***Abbreviations****: TB = Tuberculosis; HIV = Human Immunodeficiency Virus.*
