# Supplementary material for: Burden of tuberculosis in underserved populations in South Africa: A systematic review and meta-analysis
Source: PLOS Glob Public Health. 2024 Oct 3;4(10):e0003753. doi: 10.1371/journal.pgph.0003753 (PMC11449336; doi:10.1371/journal.pgph.0003753)
Supplement: S4 Fig — Abbreviations: TB = Tuberculosis; HIV = Human Immunodeficiency Virus. (DOCX) [file pgph.0003753.s014.docx]

## **S4 Fig**. Funnel plot: TB Prevalence (People living with and without HIV)

*
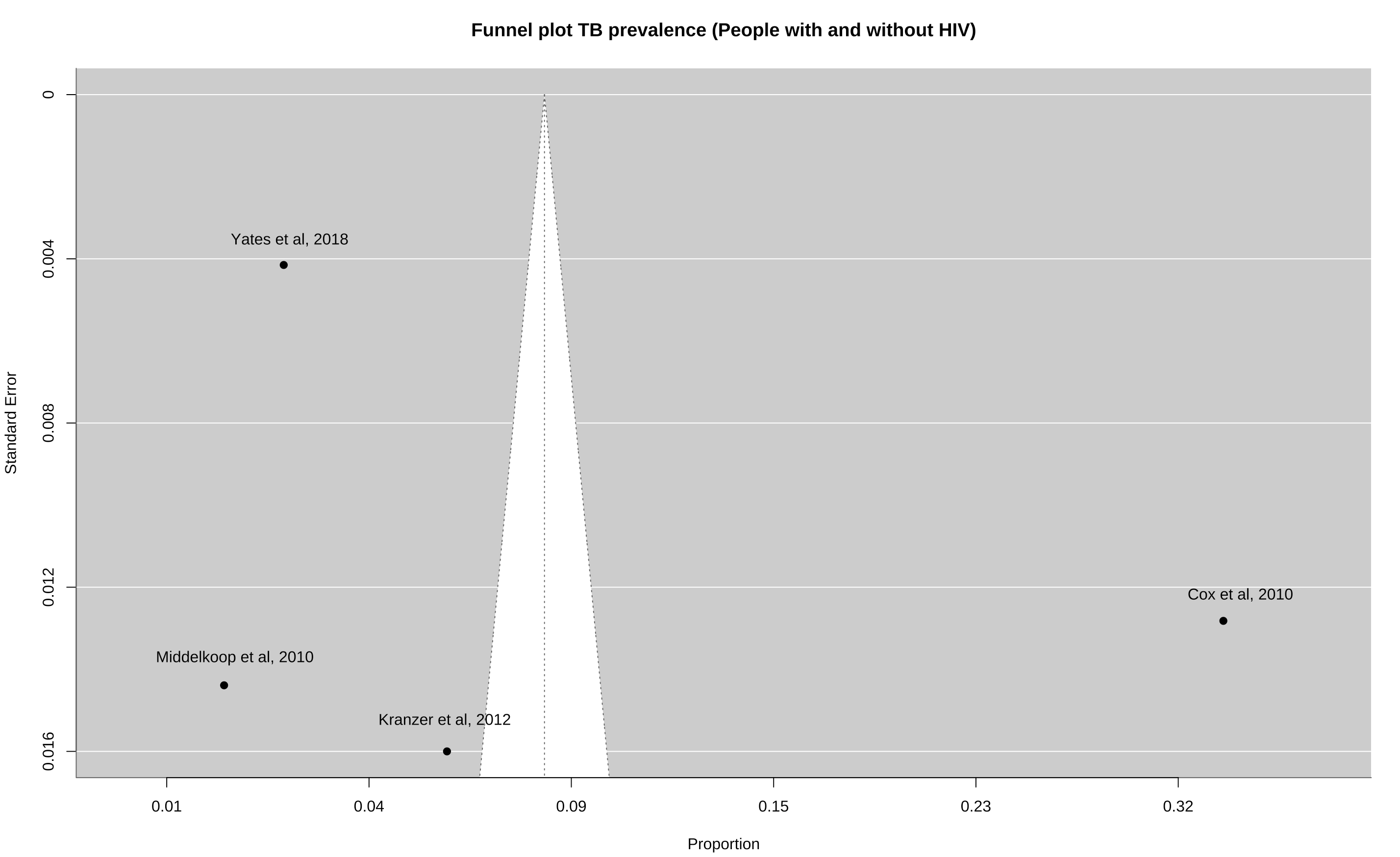
*

***S4 Fig****. Funnel plot: Pooled active TB disease prevalence among underserved populations in South Africa (‘People living with and without HIV’ subgroup).*

***Abbreviations****: TB = Tuberculosis; HIV = Human Immunodeficiency Virus.*
