# Supplementary material for: Burden of tuberculosis in underserved populations in South Africa: A systematic review and meta-analysis
Source: PLOS Glob Public Health. 2024 Oct 3;4(10):e0003753. doi: 10.1371/journal.pgph.0003753 (PMC11449336; doi:10.1371/journal.pgph.0003753)
Supplement: Attachment — Submitted filename: Rebuttal letter.pdf [file pgph.0003753.s018.pdf]

Editor's Comments to Author:

Comment 1: *Line 424 of the revised manuscript with track changes: Authors cite the article by Kubjane, M., Cornell, M., Osman, M., Boulle, A., & Johnson, L. F. (reference 63). This paper indicated that "the M:F ratios for tuberculosis incidence and mortality rates persisted above 1.0, and the figures reached 1.70 and 1.65, respectively, by the end of 2019" and further that "the 2019 estimated tuberculosis prevalence in males was 1.06% (95% CI 1.0–1.12%) and 0.58% (95% CI 0.56–0.62%) in females". These observations appear to be contradicting the authors' statement on line 424 that "..nationwide estimates, ... show higher TB prevalence and mortality in women than in men". Could this please be cross checked for consistency?*

Author's reply 1: Thank you for catching this typo. I sincerely apologize for the incorrect interpretation of the *Kubjane et al paper*'s conclusion regarding global TB mortality and incidence in men and women. In line with the paper's conclusions, we have now corrected this statement to say the following: "*Two out of four studies that reported sex-stratified TB prevalence estimates found slightly higher numbers in women than in men. This contrasts with nationwide estimates, which show higher TB prevalence and mortality in men than in women*[63]." (see line 374 - 377).

Reviewer 1' Comments to Author:

Comment 1: *I thank the authors for their diligent edits to the manuscript. The authors have addressed all my comments. The paper provides a valuable contribution to understanding the burden of TB in underserved populations in a high TB burden country.*

Author's reply 1: We appreciate your positive feedback. Thank you very much.

Reviewer 2' Comments to Author:

Comment 1: *The revised manuscript has provided satisfactory responses to the comments made in the previous review. I have only a very minor comment. On page 7 line 54 it is stated that TB*

treatment coverage (TC) increased from 57% to 77% in 2022. Could you please provide the year when the TB treatment coverage was 57% - is the baseline year 2015?

Author's reply 1: Thank you for highlighting this point. The sentence on TB treatment coverage in South Africa was part of the initial manuscript submission. However, we decided not to include it in the revised version of the manuscript. To address your query, the increase in TB treatment coverage from 57% to 77% pertains to the period between 2021 and 2022. As per the WHO,: "*(...) treatment coverage rates in South Africa rose to 77% (range: 54%-120%) in 2022—up from 57% in 2021. This is the first time that estimated treatment coverage in South Africa has exceeded 60% since the WHO began publishing data*" (source: https://www.spotlightnsp.co.za/2023/11/10/in-depth-what-new-who-tb-numbers-mean-for-sa/). I hope this answers your question.

Best regards,

Lydia Holtgrewe

Lydia Holtgrewe
