# Supplementary material for: Burden of tuberculosis in underserved populations in South Africa: A systematic review and meta-analysis
Source: PLOS Glob Public Health. 2024 Oct 3;4(10):e0003753. doi: 10.1371/journal.pgph.0003753 (PMC11449336; doi:10.1371/journal.pgph.0003753)
Supplement: Attachment — Submitted filename: Response to the Editor and Reviewers.docx [file pgph.0003753.s017.docx]

### **Editor’s Comments to Author:**

**Comment 1:** *Based on the various criteria presented in tables S4, clarify how a study was considered low vs high quality and included or excluded from the review as per table S5. Consider moving this information to the main manuscript as suggested by the reviewer.*

**Author’s reply 1 (Methods/Results)**: Thank you for this suggestion. We have now expanded on the ‘Quality Assessment’ sub-section of the Methods section based on your suggestions, replacing the term “quality” with ‘risk-of-bias’ throughout to better align with current best practices for epidemiologic research (<https://www.jclinepi.com/article/S0895-4356(20)31138-0/fulltext>):

“*We scored each item as ‘Yes’, ‘No’, ‘Unclear’ or ‘Not applicable’, resulting in an overall decision to either label studies as ‘low risk-of-bias’ or ‘high risk-of-bias’. A study’s risk of bias refers to the potential influence of its methods on the observed outcomes. Studies classified as having a high risk-of-bias are more likely to deviate in their estimates of the true effect than those classified as having a low risk-of-bias. Because of the importance of sample frame (item 1), sampling approach (item 2), and diagnostic methods (item 6) for determining prevalence and incidence in a target population, studies receiving a ‘No” response to any of these items were labelled as ‘high risk-of-bias’. (Table S4).**”* (see line 135-142).

Furthermore, we have expanded *Table S4,* *Table S5a* and *S5b* in the supplementary materials to include more detailed explanations for each of the individual assessment items and the overall appraisal received by each study. In *TableS5*, we replaced ‘Excluded/Included’ with ‘High/Low risk-of-bias’ in column M to reflect best practices and to align with the methods described in the main manuscript.

Lastly, we added a sentence to the ‘results’ section, which details why we were unable to conduct a sensitivity analysis based on the results of the risk-of-bias assessment:

‘*We were also unable to conduct sensitivity analyses based on the risk-of-bias assessment results because only one study reporting TB prevalence and only three studies reporting LTBI prevalence were considered to have a low risk of bias.*’ (see line 314-317).

**Comment 2 (Results)**: *Define what were considered "ineligible measures of disease burden"*

**Author’s reply 2**: Thank you for allowing us to clarify our intended meaning. We have rephrased ‘ineligible measures of disease burden’ as ‘ineligible study outcomes’ and added a sentence with a few examples of such outcomes, including *“(…) TB morbidity, TB mortality, and TB-related treatment costs*” (see line 187).

**Comment 3 (Methods)**: *Further, with regards to WHO endorsed diagnostic tools (line 128-129): no mention is made of the LF-LAM assay. Could the authors please clarify why this WHO endorsed diagnostic tool, and also recommended by South Africa NTP since 2021, was not included? Particularly in the view that 6 studies included in this review focused on TB in PLWH only.*

**Author’s reply 3**: Yes, the reviewer is absolutely right, we should have listed LF-LAM assays alongside other WHO-endorsed TB diagnostic tests in the ‘Data Synthesis’ section, although our search did not identify any studies that used LF-LAM assays.

### **Reviewer 1’ Comments to Author:**

**Comment 1 (Introduction):** *Lines 51–56: The authors mention the WHO End TB Targets. For readers who are unfamiliar, the authors should briefly define the targets.*

**Author’s reply 1**: Yes, this is a good suggestion. We have now defined the WHO End TB Targets and interim milestones in the Introduction, and also highlighted South Africa’s progress towards attaining those targets and milestones:

“*The 2015 End TB Strategy proposed ambitious targets to reduce TB incidence by 80% and TB deaths by 90% by 2030^[2]^. While cumulative global reductions in these indicators fell short of the 2020 interim milestones^[1-3]^, South Africa reported a 53% reduction in incidence between 2015 and 2022, from 988 to 468 persons with TB per 100,000 population, although the TB mortality has not fallen as rapidly. Novel strategies are needed to reach the WHO targets* ^[4]^*.*” (see line 52-57).

**Comment 2 (Introduction):** *Line 52: Consider also citing the latest Global Burden of Disease report illustrating that the End TB targets were not achieved at the global level.*
[*https://pubmed.ncbi.nlm.nih.gov/38518787/*](https://eur03.safelinks.protection.outlook.com/?url=https%3A%2F%2Fpubmed.ncbi.nlm.nih.gov%2F38518787%2F&data=05%7C02%7Clmlh1g18%40soton.ac.uk%7C48284a97438f48dd02ce08dc6e116315%7C4a5378f929f44d3ebe89669d03ada9d8%7C0%7C0%7C638506268174950935%7CUnknown%7CTWFpbGZsb3d8eyJWIjoiMC4wLjAwMDAiLCJQIjoiV2luMzIiLCJBTiI6Ik1haWwiLCJXVCI6Mn0%3D%7C0%7C%7C%7C&sdata=HOOjH5O9z4Zhr%2F34ar7%2BlcSo8c%2FrhSq0fVwobXIzMt0%3D&reserved=0)

**Author’s reply 2**: We agree that this would be helpful and have now cited the 2021 Global Burden of Disease Report alongside the End TB Strategy document (see references [2] and [3]).

**Comment 3 (Introduction*):*** *Please use PLWH for people living with HIV rather than PWH*.

**Author’s reply 3**: Thank you for pointing this out. We have replaced ‘PWH’ with ‘PLWH’ throughout the manuscript. We have also replaced the term ‘TB cases’ with ‘people with TB’ throughout the manuscript to reflect the latest guidance from TB advocacy groups on avoiding stigmatizing language.

**Comment 4 (Methods):** *Lines 86 – 88: Why wasn’t PubMed also queried? This is important because prior work has shown that a combination of Embase and PubMed yields high coverage.*
[*https://pubmed.ncbi.nlm.nih.gov/29208034/*](https://eur03.safelinks.protection.outlook.com/?url=https%3A%2F%2Fpubmed.ncbi.nlm.nih.gov%2F29208034%2F&data=05%7C02%7Clmlh1g18%40soton.ac.uk%7C48284a97438f48dd02ce08dc6e116315%7C4a5378f929f44d3ebe89669d03ada9d8%7C0%7C0%7C638506268174956830%7CUnknown%7CTWFpbGZsb3d8eyJWIjoiMC4wLjAwMDAiLCJQIjoiV2luMzIiLCJBTiI6Ik1haWwiLCJXVCI6Mn0%3D%7C0%7C%7C%7C&sdata=e2TLTst4mdV3HKpjxABnWb9gNVrFG%2F%2Bj6krqCyQbVSw%3D&reserved=0)

**Author’s reply 4:** Good question, thank you. Our primary search database, *Lens.org*, aggregates sources from multiple other databases, including *PubMed, Microsoft Academic, Crossref,* and many open access journals based in low-income economies. Because of its wide scope and coverage of local South African journals, we chose it over PubMed/Medline only. We have now included a sentence describing Lens.org in the Methods:

“*Lens.org aggregates content from multiple sources including PubMed, Microsoft Academic, and Crossref, and helps identify free full-text versions of papers*.” (see line 89-91).

**Comment 5 (Methods*):*** *Study selection: Please include additional information for inclusion criteria such as types of study designs were included, sampling methods, populations, outcome measurement, etc. This is important because I’m very unclear on the exact inclusion criteria for the systematic review.*

**Author’s reply 5**: We added a few sentences to the ‘Study selection’ section to clarify the inclusion criteria:

“*We included 1) peer-reviewed articles and pre-print manuscripts of prospective and retrospective cohort studies, cross-sectional studies, non-randomized studies and randomized controlled trials that were 2) published or made available between 1 January 2010 and 26 July 2023, regardless of language. We required studies to 3) report prevalence, incidence, or notification data on active TB disease or LTBI, and to include 4) underserved populations in South Africa, including townships, informal settlements, and impoverished populations. To guide screening of articles, we defined townships as tightly regulated, racially segregated residential areas built outside cities during the Apartheid Era. We defined informal settlements as residential areas constructed on land that occupants have no legal claim to occupy, often in the context of rapid population growth and an inadequate housing supply. Both are characterized by substandard living conditions^[20,21]^. We defined impoverished communities as those consisting of individuals of low socio-economic status, characterised by low household income^[22]^. Because studies reported wealth and income in different ways, we classified a study population as impoverished if the study referenced any measure or proxy indicative of low household wealth or income.”* (see line 101-115).

We did not specify sampling methods as an inclusion criterion, but rather assessed this parameter as part of the risk-of-bias assessment.

**Comment 6 (Methods):** *Quality assessment: Please include 2-3 sentences on which metrics were used to evaluate study quality and why. Please also reference Table S4 here to point to additional details for readers. In addition, what is considered to be high-quality vs low-quality?*

**Author’s reply 6**: Please refer to ‘Author’s Reply 1’ to the Editor’s Comment 1 above to see how we have addressed this point.

**Comment 7 (Methods*):*** *Sensitivity analyses: Was it possible to conduct sub-group analyses by sex? It may be interesting to see if the male to female ratio among underserved population differs compared to other estimates.*

**Author’s reply 7**: We agree that stratifying our results by sex would be both interesting and highly relevant. Unfortunately, only three of the eight studies in the meta-analysis of TB prevalence and one of the five studies in the meta-analysis of LTBI prevalence reported TB status by sex. Consequently, we were unable to conduct subgroup meta-analyses by sex. Nevertheless, we have included statements reporting sex-stratified outcomes for the studies that provided this information in the ‘Results’ and ‘Discussion’ sections:

**Results section**:

**TB prevalence**: ‘*Lastly, four studies reported TB prevalence stratified by sex, ranging from 3.4%^[37]^ to 34.8%^[35]^ in women and from 6.1%^[36]^ to 29.2%^[35]^ in men*.’ (see line 248-250).

**LTBI prevalence**: ‘*One study reported LTBI prevalence by sex, finding a LTBI prevalence of 32.3% in women and 37.1% in men^[47]^*.’ (see line 254-255).

**TB incidence**: ‘*One study reported sex-stratified TB incidence rates, finding a TB incidence of 2.4 people with TB per 100 person-years in women and 3.5 people with TB per 100 person-years in men^[53]^.*’ (see line 268-270).

**LTBI incidence**: ‘*Of the two LTBI incidence studies, one was conducted in children and reported an incidence rate of 11.8 people with TB per 100 person-years overall, 9.4 people with TB per 100 person-years in girls, and 14.3 people with TB per 100 person-years in boys using TST. ^[53]^’* (see line 272-275).

**Discussion section**: ‘*Subgroup analyses based on sex and age would have been particularly relevant due to significant variations in LTBI prevalence across different age groups and between men and women. Two out of four studies that reported sex-stratified TB prevalence estimates found slightly higher numbers in women than in men. This contrasts with nationwide estimates, which show higher TB prevalence and mortality in women than in men^[63].^ All other studies that provided sex-specific outcomes reported lower prevalence and incidence estimates of active TB disease and LTBI in women compared to men.*’ (see line 369-375).

**Comment 8 (Methods):** *Other effect estimates: Have the authors considered using estimates from the Global Burden of Disease study as the comparator for the overall national-level TB prevalence in South Africa?*
[*http://ihmeuw.org/6e62*](https://eur03.safelinks.protection.outlook.com/?url=http%3A%2F%2Fihmeuw.org%2F6e62&data=05%7C02%7Clmlh1g18%40soton.ac.uk%7C48284a97438f48dd02ce08dc6e116315%7C4a5378f929f44d3ebe89669d03ada9d8%7C0%7C0%7C638506268174962532%7CUnknown%7CTWFpbGZsb3d8eyJWIjoiMC4wLjAwMDAiLCJQIjoiV2luMzIiLCJBTiI6Ik1haWwiLCJXVCI6Mn0%3D%7C0%7C%7C%7C&sdata=k77Jr4tsTUTtEBYNPSZ3Tdm%2BAcmP8xM3y%2B0AHy3RTD0%3D&reserved=0)

**Author’s reply 8**: Thank you for this suggestion which we have explored in detail. We extracted the GBD study’s TB prevalence estimate for 2018 and compared it to the estimate from the First National TB Prevalence Survey (2018), which we used in our analysis. The GBD estimate is only slightly higher than that of the First National TB Prevalence Survey (**0.79% vs 0.74% in 2018**). Using the GBD estimate would not meaningfully change our relative risk estimates for underserved populations in South Africa. Therefore, the choice of national prevalence input estimates likely does not affect our findings.

**Comment 9 (Results*):*** *Lines 169: What are ineligible measures of disease burden?*

**Author’s reply 9**: Please refer to ‘Author’s reply 2’ to the Editor’s comment 2 above to see how we have addressed this point.

**Comment 10 (Results):** *Table 1: The authors mention that many studies are not population-based but recruited. This is an important point. Can the authors include an additional column in Table 1 for recruitment strategy/sampling?*

**Author’s reply 10:** That is a great idea. We added an additional column to *Table 1* with the requested information on the study’s sampling strategy. This is also addressed in the risk-of-bias assessment section and supplementary materials (*Table S5a, S5b*).

**Comment 11 (Discussion):** *Lines 367–376: Can the authors comment on previous interventions that have been shown to improve case finding and TB outcomes among underserved populations. Since the goal of the study was to help inform the national TB program in South Africa, this provides valuable insights.*

**Author’s reply 11**: We have added a few sentences on previous studies on active case-finding strategies conducted in South African informal settlements and townships in recent years, such as making novel, highly sensitive, point-of-care diagnostic tools available in mobile clinics positioned in or near underserved communities, and the use of targeted case-finding strategies among household contacts of TB index cases to increase case detection in high-prevalence settings:

“*To effectively curb these figures, it is imperative to implement a diverse array of testing modalities that are acceptable, feasible, cost-effective, and can be seamlessly integrated into targeted case-finding initiatives that focus on ‘hard-to-reach’ populations^[71-74]^. For example, a community-based active case-finding initiative using rapid point-of-care (POC) GeneXpert testing in mobile clinics within peri-urban informal settlements of Cape Town has shown promise, by decreasing time to treatment initiation and increasing the percentage of people receiving TB treatment compared to facility-based testing using sputum smear microscopy^[75]^. Strategies like these may be especially beneficial in informal settings where laboratory facilities and qualified medical personnel are scarce, by helping reach individuals who do not present to healthcare facilities^[76,77]^. The National TB Strategic Plan 2023-28 identifies informal settlements as a priority population for novel POC diagnostic tools, such as digital chest x-ray, tongue swabs, and self-screening. Additionally, it notes that treatment modalities like community-based care and adherence support should be considered, bolstered by laboratory data and geo-spatial mapping^[78]^*.” (see line 427-439).

**Comment 12 (Discussion):** *Can the authors briefly comment how the COVID-19 pandemic may have impact TB burden among underserved populations in South Africa?*

**Author’s reply 12**: Yes, this is an important topic. We have included two sentences on what is known about the potential impacts of the COVID-19 pandemic on the TB burden in underserved populations in South Africa:

‘*Lastly, data on the impacts of the COVID-19 pandemic on the TB burden in underserved populations is scarce^[64]^. These populations face increased vulnerability to the pandemic’s consequences due to a loss of household income without a financial safety net and poor access to social assistance programmes, testing, and healthcare services^[65]^*’ (see line 385-389).

### **Reviewer 2’ Comments to Author:**

**Comment 1 (Strengths):** *Strengths: The review is of high quality and conforms with accepted norms for the conduct of a systematic review and meta-analysis including in methodological approach, literature search strategy, assessment of bias, measures/ascertainment of outcomes etc.*

**Author’s reply 1**: Thank you for your encouraging feedback, Prof. Dr. Chakaya. We have addressed your suggestions for improving the quality of our manuscript below.

**Comment 2 (Weaknesses – Introduction):** *A description of the burden of TB in South Africa, including incidence and prevalence is not provided to allow readers of this paper to have a better understanding of the TB situation in this country. The introduction only includes a statement about TB treatment coverage in South Africa (line 52-55).*

**Author’s reply 2**: Thank you for highlighting this. To enable the reader to better understand the current TB situation in South Africa, we have now included up-to-date TB incidence estimates, and explained how these relate to TB treatment coverage and the End TB Strategy’s targets:

“*The 2015 End TB Strategy proposed ambitious targets to reduce TB incidence by 80% and TB deaths by 90% by 2030^[2]^. While cumulative global reductions in these indicators fell short of the 2020 interim milestones^[1-3]^, South Africa reported a 53% reduction in incidence between 2015 and 2022, from 988 to 468 persons with TB per 100,000 population, although the TB mortality has not fallen as rapidly. Novel strategies are needed to reach the WHO targets ^[4]^*” (see line 52-57).

**Comment 3 (Weaknesses - Methods):** *While the review was focused on underserved” populations including people living in informal settlements, townships and impoverished communities only the population of people living in informal settlements is clearly defined (Lines 101-103). It is not clear if living in informal settlement is the same as living in a township. The measure of impoverishment in South Africa is also not defined.*

**Author’s reply 3**: We appreciate the suggestion to clarify our use of these terms. We have expanded on the definition of townships, informal settlements and impoverished populations in the ‘Study Selection’ section:

“*To guide screening of articles, we defined townships as tightly regulated, racially segregated residential areas built outside cities during the Apartheid Era. We defined informal settlements as residential areas constructed on land that occupants have no legal claim to occupy, often in the context of rapid population growth and an inadequate housing supply. Both are characterized by substandard living conditions^[20,21]^. We defined impoverished communities as those consisting of individuals of low socio-economic status, characterised by low household income^[22]^. Because studies reported wealth and income in different ways, we classified a study population as impoverished if the study referenced any measure or proxy indicative of low household wealth or income*.” (see line 107-115).

We have also included the definitions of ‘impoverished populations’ used in these two studies in the legend of *Table 1.*

**Comment 4 (Weaknesses - Discussion):** *It is noted that included studies were mostly carried out among township populations (9 of 12 studies for prevalence of active TB, 4 out of 5 studies for LTBI prevalence, 5 of 5 studies for TB incidence and 2 of 2 studies for LTBI incidence) and mostly in Cape Town. While the authors acknowledge this limitation, there is no apparent push or argument to conduct research studies similar to those included in this systematic review in other regions of the country and also in other populations considered at risk for TB (for example rural poor populations, people deprived of liberty and others). It is therefore uncertain how the results of this study will inform the development of South Africa's National TB Strategy.*

**Author’s reply 4**: Thank you for this useful comment. While we had already included a sentence on the need to conduct similar TB prevalence/incidence studies in regions other than Cape Town, we have now expanded on this recommendation to include other at-risk populations as the Reviewer suggests. Additionally, we have included a statement on how our systematic review impacted the National TB Strategic Plan 2023-2028:

“*Further research is needed not only in different geographical areas but also among other at-risk populations, including but not limited to rural poor communities, people who are incarcerated, and refugee and migrant populations^[14]^*” (see line 379-381).

“*The results from our analysis have contributed to updated TB prevalence estimates for people living in informal settlements in South Africa's National TB Strategic Plan 2023-2028^[67]^”* (see line 422-423).

**Comment 5 (Weaknesses - Discussion):** *The “global value” of the results obtained by this systematic review and meta-analysis appears limited – what this systematic review and meta-analysis reveals has been known for a long time.*

**Author’s reply 5**: We acknowledge the Reviewer’s point, although given the dramatic decreases in TB incidence in South Africa in recent years, we did not know that this would be the case before undertaking the review. As noted above, rigorously reviewing and summarizing the recent data was an important step in developing the revised National TB Strategic Plan for 2023-2028. Specifically, the findings have shaped the Plan to emphasize the need for case finding and prevention interventions in underserved communities, the importance of which is expected to continue to grow in the context of ongoing migration, urbanization, and inflation in South Africa. Finally, we identify methodological flaws and gaps in existing research on the TB burden among underserved populations, thereby highlighting areas where further research is needed to better understand the spatial epidemiology of TB.
